# Mothers’ Body Appreciation and Postpartum Self-Esteem in Relation to Body Changes and Breastfeeding Difficulties: A Cross-Sectional Survey in Cyprus

**DOI:** 10.3390/nursrep15030076

**Published:** 2025-02-24

**Authors:** Anastasia Kalli, Maria Iliadou, Ermioni Palaska, Sevasti Louverdi, Calliope Dagla, Eirini Orovou, Maria Dagla

**Affiliations:** 1 Laboratory of Midwifery Care During Antenatal and Post Natal Period—Breastfeeding, Department of Midwifery, School of Health and Care Sciences, University of West Attica, 12243 Athens, Greece; anastasia.kalli4@gmail.com (A.K.); miliad@uniwa.gr (M.I.); epalaska@uniwa.gr (E.P.); slouverdi@uniwa.gr (S.L.); kelkeldag@gmail.com (C.D.); 2 Department of Midwifery, School of Health and Care Sciences, University of Western Macedonia, 54636 Kozani, Greece; eorovou@uowm.gr

**Keywords:** self-esteem, body appreciation, breastfeeding, postpartum, breastfeeding difficulties

## Abstract

**Objectives**: This study explores the effect of pregnancy weight gain, acceptance of body changes, and breastfeeding difficulties on mothers’ self-esteem and body appreciation during the postpartum period. **Methods**: A cross-sectional survey was conducted among 100 puerperae (at 2nd day postpartum), during August 2022–March 2023 in Paralimni/Cyprus, with exclusion criteria being the presence of postpartum depression. The Body Appreciation Scale—BAS—was administrated in order to investigate the level of body appreciation, and the Rosenberg Self-Esteem Scale—RSES-10—was used for assessing self-esteem. **Results**: Findings indicated that the higher the pregnancy weight gain (*r* = −0.293), the lower the measured levels of the mothers’ self-esteem and body appreciation are (*r* = −0.292). Mothers whose level of acceptance of body changes was low to moderate appeared to demonstrate lower self-esteem (*p* = 0.027) and lower body appreciation (*p* < 0.001) at two days postpartum. Also, mothers who had breastfeeding difficulties at two days postpartum seem to demonstrate lower self-esteem (*p* = 0.032), and increased support from their husbands in terms of breastfeeding is associated with higher levels of mothers’ self-esteem (*r* = 0.235). Additionally, greater support for breastfeeding, both in the clinic (*r* = 0.314) and from their husbands (*r* = 0.290), appears to be linked to higher levels of body appreciation. The psychological influence of pregnancy weight gain (*β* = −0.236, *p* = 0.04) and breastfeeding difficulties (*β* = −0.284, *p* = 0.008) appeared to be predictors of mothers’ self-esteem at two days postpartum. **Conclusions**: The findings highlight the need for further research in areas including mothers’ body image and self-esteem and breastfeeding difficulties and further longitudinal studies to determine the exact nature of the pathways involved.

## 1. Introduction

The World Health Organization (WHO) recommends that breast milk should be provided exclusively to infants up to six months, followed by complementary breastfeeding until the age of two [1]. This guideline is based on various research, with documented positive results of breastfeeding for the health of mother, child, and society at the same time [2,3,4]. In the research of Victora and colleagues [5], it has been shown that if breastfeeding is implemented as recommended, from the birth of the infant up to the age of two, then it is very likely to prevent more than 800,000 deaths in children under the age of five at a global level. Through a worldwide survey completed in 2019 by the World Health Organization during the period from 2013 to 2018, 41% of infants under six months were exclusively breastfed [6]. Nevertheless, body image concerns have been widely documented as a reason why most women reduce the breastfeeding duration [7,8,9].

Negative body image concerns among breastfeeding mothers may lead to depressive symptoms. Such symptoms include constant sadness, general indifference to the environment, loss of appetite, and reduced self-esteem. These factors may lead to shorter breastfeeding durations [10,11,12]. Research also shows that body image concerns are associated with eating disorders among breastfeeding mothers during the postpartum period and may lead to maternal obesity [7,13]. Breastfeeding mothers often feel that they are unattractive or overweight. Worrying about obesity, and all these negative emotions, can lead mothers to stop breastfeeding at an early stage [13]. There are some mothers who, due to their weight gain during pregnancy, may not want at all to breastfeed their infants [8,14]. On the other hand, there are women who admire their body image during breastfeeding [15]. This element may be related to the fact that young mothers may lose weight during the breastfeeding period [16].

Lovering and colleagues [17], in their research, have shown that some breastfeeding mothers are vulnerable to the sociocultural pressure to lose weight immediately after childbirth. Unrealistic representations of women through various media can also increase pressure on mothers to lose weight while breastfeeding [18]. Celebrities and some mothers portray postpartum weight loss as automatic and easy. Unfortunately, such interpretations may pressure new mothers to lose weight immediately after giving birth [17], together with the frequent pressure of partners, family members, colleagues, and peers.

The WHO has established a target to achieve a 70% exclusive breastfeeding rate for infants up to the age of 6 months by the year 2030 [6]. Health professionals can influence the mother’s decision and attitude towards breastfeeding [19]. Both nurses and midwives play a fairly important role in the choice a mother makes for her infant’s nutrition. Fathers of infants also play an important role in supporting and promoting breastfeeding by providing emotional support and participating in infant feeding decisions [20]. Overall, breastfeeding should be considered a public health issue, to be addressed holistically by society [21,22].

It is critical to understand the factors that promote breastfeeding in order to address low breastfeeding rates and duration [22]. Breastfeeding duration has been shown to be influenced by maternal psychological differences, such as the mother’s degree of self-esteem and her experiences with stress, depression, and anxiety [23]. The mother’s self-esteem is thus one of the psychological factors that can help her cope with stress and support successful and ongoing breastfeeding [23]. Two qualitative studies showed that breastfeeding duration was significantly related to maternal self-esteem, while others found that primiparous women’s lack of experience, knowledge, and skills in breastfeeding led to mothers feeling anxious about breastfeeding and having low self-esteem [24]. Early on after giving birth, these mothers with low self-esteem may have a negative experience with motherhood, being unable to adequately breastfeed and care for their child [25]. In their research, Mannion and coworkers found that lactating women had high levels of self-esteem, which were associated with higher levels of self-efficacy [26].

In Cyprus and Greece, there are no studies regarding mothers’ self-esteem and body appreciation in relation to body changes postpartum and breastfeeding difficulties. Therefore, the purpose of the current study is to investigate the effect of pregnancy weight gain, acceptance of body changes and breastfeeding difficulties on mother’s self-esteem and body appreciation at postpartum period.

## 2. Materials and Methods

### 2.1. Study Population

One hundred women who have given birth in Paralimni/Cyprus, during the period August 2022–March 2023 in a private hospital took part in this research. In order to be a part of the current study, women had to: (a) be older than 18 years, (b) give birth in clinics, (c) show intention to participate, and (d) have the ability to complete questionnaires. It is addressed to mothers of both natural birth and cesarean section, who are breastfeeding, will be breastfeeding, or not breastfeeding. Exclusion criteria were: (a) women who did not intend to participate in the research and (b) women with postpartum depression. This study was approved by the Research Ethics Committee of the private hospital in Paralimni/Cyprus (Ref.7/2022).

### 2.2. Procedure

A cross-sectional survey was conducted among 100 puerperae (on the 2nd day postpartum) who expressed their interest in participating in the study; they were given the Research Information Note, as well as the Consent to be signed, prior to their participation. Apart from the study process, they were informed that their participation would be anonymous and voluntary, as well as about the possibility to withdraw from the research at any time. All the participants complete a questionnaire and two psychometric scales during the 2nd day after delivery, through personal contact with the researcher.

### 2.3. Measures

Within information collected from participants, on the 2nd day postpartum, is information referring to demographic characteristics, certain perinatal characteristics, and breastfeeding characteristics (questionnaire). Also, all participants filled out the psychometric scales below:

*Body Appreciation Scale—BAS* [27] assesses acceptance and/or favorable opinions towards the body through 13 self-report items. The items are rated on a 5-point Likert-type scale ranging from 1 “never” to 5 “always”. Items were created specifically to measure how much women: (a) have positive body perceptions, (b) accept their bodies despite their weight, body type, and flaws, (c) respect their bodies by taking care of their needs, and (d) protect their body image by rejecting unrealistic images of the thin-ideal prototype portrayed in the media. These characteristics show complete acceptance and reverence for the body, a component of good body image that is called “body appreciation”. The range of scores is 0–30, with higher scores indicating higher body appreciation. Cronbach’s α score for these items was found to be 0.93; in this study, the reliability index was 0.80.

*Rosenberg Self-Esteem Scale—RSES-10* [28] captures the global perception of the individual’s self-worth through a 10-item tool. All items are answered using a 4-point Likert scale format ranging from 1 “strongly agree” to 4 “strongly disagree”. The range of scores is 0–30, with higher scores indicating higher self-esteem. Cronbach’s α reliability index values range from 0.72 to 0.87; the reliability index reached 0.91 in the present study.

## 3. Results

The sample of the present study was 100 new mothers with a mean age of 30.92 ± 4.83 (*SD*) years. The majority of them were married (*n* = 81, 81.0%). The monthly income was 1000–2500 euro for approximately half of the participants (*n* = 49, 49.0%), with most of them (*n* = 81, 81.90%) having completed at least undergraduate studies. The mean of the sample’s weight before pregnancy was 66.52 ± 16.75 (*SD*), while during the pregnancy, weight gain was 12.8 ± 5.27 (*SD*). Half of the women gave birth with prearranged cesarean section (*n* = 51, 51.0%) while only 23.0% (*n* = 23) of them gave birth by vaginal delivery. Breastfeeding information was obtained for the majority of them (*n* = 71, 71.0%), and half of them had decided to breastfeed before pregnancy (*n* = 50, 50.0%). Most of them received significant breastfeeding support in the clinic (*n* = 80, 80.0%) and from their husbands (*n* = 73, 73.0%). However, 31.0% (*n* = 31) of the sample faced breastfeeding difficulties in the following days after delivery, while 32.0% (*n* = 32) of them had difficulties accepting their body changes.

Before analyzing the relationship between mothers’ body appreciation and self-esteem and breastfeeding, two analyses were conducted in order to examine if the pregnancy weight gain appears to manifest any connection between mother’s self-esteem and body appreciation postpartum. Table 1 presents the results of the bivariate Pearson’s *r* correlations analysis among self-esteem and body appreciation at two days postpartum and pregnancy weight gain.

The correlation analysis showed statistically significant, negative, moderate correlations between both self-esteem and pregnancy weight gain (*r* = −0.293) and body appreciation and pregnancy weight gain (*r* = −0.292) at two days postpartum. It appears that the higher the pregnancy weight gain, the lower the measured levels of mother’s self-esteem and body appreciation among the participants in this study. Next, the results of multivariate analyses of variance of mother’s self-esteem and body appreciation at two days postpartum in relation to the psychological influence of pregnancy weight gain are presented in Table 2.

At the univariate level, the *F* criterion showed a statistically significant relationship between mother’s self-esteem and body appreciation at two days postpartum in relation to the psychological influence of pregnancy weight gain. According to the *Scheffé* post hoc criterion, it is observed that mothers of this sample, whose psychological influence of pregnancy weight gain was moderate to high, are more likely to have lower self-esteem at two days postpartum, in relation to those whose psychological influence of pregnancy weight gain was absent (*p* = 0.015), with 8.3% of the variance explained. Similarly, those mothers whose psychological influence of pregnancy weight gain was moderate to high seem to express lower body appreciation at two days postpartum in relation to those whose psychological influence of pregnancy weight gain was absent (*p* < 0.001), with 15.1% of the variance explained. Table 3 presents the results of multivariate analyses of variance of self-esteem and body appreciation at two days postpartum in relation to the level of acceptance of body changes.

Yet again, at the univariate level, the *F* criterion showed a statistically significant relationship between mothers’ self-esteem and body appreciation at two days postpartum in relation to the level of acceptance of body changes. The *Scheffé* post hoc criterion revealed that mothers of this sample, whose level of acceptance of body changes was low to moderate, appeared to demonstrate lower self-esteem at two days postpartum, in relation to those whose level of acceptance of body changes was very high (*p* = 0.027), with 7.2% of the variance explained. Correspondingly, those mothers whose level of acceptance of body changes was low to moderate, demonstrated lower body appreciation at two days postpartum, in relation to those whose level of acceptance of body changes was high to very high (*p* < 0.001), with 19.0% of the variance explained.

After evaluating the relationship between mother’s self-esteem and body appreciation in association with pregnancy weight gain and body changes variables, the aim of the following analyses is to examine if there is any association between mothers’ self-esteem and body appreciation and breastfeeding variables. First, such results are presented in Table 4, which emerged from conducted multivariate analyses of variance conducted on mothers’ self-esteem and body appreciation two days postpartum, in relation to breastfeeding difficulties. These difficulties were measured by the item: “At this moment, to what extent are you experiencing difficulties while breastfeeding”? Responses were rated on a 5-point Likert scale, ranging from 1 (“not at all”) to 5 (“very much”).

At the univariate level, the *F* criterion showed a statistically significant relationship between self-esteem and body appreciation at two days postpartum in relation to breastfeeding difficulties. The results show that those who had breastfeeding difficulties at two days postpartum seem to demonstrate lower self-esteem (*p* = 0.032), with 5.5% of the variance explained, while there was no relation to body appreciation.

Table 5 presents the results of the bivariate Pearson’s *r* correlations analysis among mothers’ self-esteem and body appreciation at two days postpartum and breastfeeding support in hospital, measured by the items: “Is there a support you would like for breastfeeding now that you are in the hospital”?, “To what extent do you feel that your husband supports you in breastfeeding”? and “To what extent do you feel that your midwives support you in breastfeeding”? Responses were rated on a 5-point Likert scale, ranging from 1 (“not at all”) to 5 (“very much”). For the analysis, these three items about breastfeeding support were computed and mean scores were used.

The correlation analysis showed statistically significant, positive, low to moderate correlations between mothers’ self-esteem and breastfeeding support from the husband (*r* = 0.235), and positive, moderate correlations among body appreciation and breastfeeding support in the clinic (*r* = 0.314) and from the husband (*r* = 0.290) at two days postpartum. It appears that increased support from husbands in terms of breastfeeding is associated with higher levels of self-esteem in the sample. Additionally, greater support for breastfeeding, both in the clinic and from husbands, appears to be linked to higher levels of body appreciation.

The next step was to check on the possible regression model of prediction, including all the above independent variables in relation to mother’s self-esteem and body appreciation at two days postpartum. Table 6 presents the results of linear regression analyses, in terms of variables of pregnancy weight gain, psychological influence of pregnancy weight gain, level of acceptance of body changes, breastfeeding difficulties, and breastfeeding support.

Through linear regression, with self-esteem being the dependent variable, in the current regression model, only psychological influence of pregnancy weight gain (*β* = −0.236, *p* = 0.04) and breastfeeding difficulties (*β* = −0.284, *p* = 0.008) appeared to be predictors of self-esteem at two days postpartum. The percent of variability interpreted by this model is statistically different from 0, with the regression equation reaching significance (*F* = 3.688, *df* = 6, *p* = 0.003, *R* = 0.475, and *R*^2^ = 0.226), with 22.6% of the variance explained. Accordingly, this analysis revealed that higher self-esteem at two days postpartum is associated with less psychological influence of pregnancy weight gain and fewer breastfeeding difficulties.

In the regression model relating body appreciation predictors were the psychological influence of pregnancy weight gain (*β* = −0.262, *p* = 0.013), level of acceptance of body changes (*β* = 0.232, *p* = 0.045), and breastfeeding support in the clinic (*β* = 0.236, *p* = 0.048). The percent of variability interpreted by this model is statistically different from 0, with the regression equation reaching significance (*F* = 7.100, *df* = 6, *p* < 0.001, *R* = *0*.0599 and *R*^2^ = 0.359), with 35.9% of the variance explained. It appeared that higher body appreciation at two days postpartum is associated with less psychological influence of pregnancy weight gain, a higher level of acceptance of body changes and more breastfeeding support in the clinic. Finally, Table 7 presents the results of linear regression analysis, examining the influence of information on the initiation and continuation of breastfeeding in relation to the degree of psychological influence of pregnancy weight gain.

In the last analysis of linear regression, the predictor for the degree of psychological influence of pregnancy weight gain was the influence of information on the initiation and continuation of breastfeeding (*β* = −0.345, *p* = 0.042). The percent of variability interpreted by this model is statistically different from 0, with the regression equation reaching significance (*F* = 10.283, *df* = 1, *p* = 0.002, *R* = 0.354 and *R*^2^ = 0.108), with 10.8% of the variance explained. Therefore, it showed that those who were more influenced by information on initiation and continuation of breastfeeding seem to demonstrate lower degrees of psychological influence of pregnancy weight gain.

## 4. Discussion

The current study sought to explore the effect of pregnancy weight gain, body changes, and breastfeeding difficulties on mothers’ self-esteem and body appreciation during the postpartum period. The findings highlighted several key relationships: breastfeeding difficulties were significantly negatively associated with both mothers’ self-esteem and body appreciation. Conversely, breastfeeding support in the hospital, as well as from husbands, was positively correlated with both higher self-esteem and better body appreciation. Additionally, breastfeeding difficulties emerged as a predictor of mothers’ self-esteem at two days postpartum, while breastfeeding support from healthcare professionals in the hospital predicted mothers’ body appreciation. Moreover, the provision of information on breastfeeding initiation and continuation was found to influence the psychological impact of pregnancy weight gain.

Mothers’ perceptions of their new role as a “food provider” for their infant likely contribute to this positive relationship between breastfeeding and self-esteem. Fabian and colleagues found that low self-esteem during lactation was associated with insufficient lactation experience, knowledge, and skills, which aligns with our results showing a positive relationship between self-esteem and hospital-based breastfeeding support [29]. Further supporting the importance of self-esteem in breastfeeding, research has shown that maternal self-esteem at four weeks after birth and partner support significantly predicts breastfeeding outcomes, including rates of exclusive breastfeeding and comorbidity rates [30,31]. Educational interventions aimed at enhancing self-esteem are essential, as they can be tailored to support mothers in overcoming breastfeeding difficulties, regardless of their socioeconomic background [32,33]. Such interventions can guide healthcare professionals in offering the necessary support, ultimately improving breastfeeding outcomes and maternal well-being.

Our findings on the relationship between body appreciation and breastfeeding align with earlier studies that highlighted the impact of body image issues on both antepartum and postpartum experiences [8,34,35]. These associations may be explained by a shift in participants’ perceptions of their bodies, where focusing more on their body’s functional capabilities—such as giving birth and nourishing their baby—reduces the emphasis on appearance [35,36,37]. This shift can also help mothers cope with breastfeeding difficulties, as a more positive body image may foster greater confidence in their ability to breastfeed, even when challenges arise. When mothers value their bodies for their functional strengths, they may be better equipped to overcome obstacles like pain, lactation issues, or initial breastfeeding difficulties, ultimately enhancing breastfeeding self-efficacy.

The work of Rodgers and colleagues emphasizes the importance of including maternal body image and eating issues in psychoeducational programs designed to promote breastfeeding [38,39]. Such programs should provide mothers with realistic expectations regarding postpartum weight loss and body image. Schalla, Witcomb, and Haycraft [17] also found that many mothers lack an understanding of how breastfeeding can aid in regaining their pre-pregnancy weight and shape, as well as provide other functional benefits. Increasing awareness of these benefits may help reduce body-image-related barriers to breastfeeding. These findings suggest that programs addressing body dissatisfaction, including reasonable weight-loss expectations, could benefit mothers feeling pressure from unrealistic beauty standards during the postpartum period [17]. The implications of these findings suggest that interventions focusing on both breastfeeding and body image should be incorporated into healthcare services for new mothers. For example, psychoeducational workshops could be designed to inform mothers about the benefits of breastfeeding for both their bodies and their babies. These workshops could include discussions about body image, self-esteem, and strategies to manage societal pressures surrounding postpartum appearance. Healthcare providers should also be trained to address body image issues and provide psychological support alongside practical breastfeeding guidance.

One limitation of this study is the small sample size, which may not be representative of the broader Cyprus puerperae population. As such, the generalizability of these findings may be limited. Future studies should include larger, more diverse populations, including mothers from various socioeconomic backgrounds and cultural contexts. This would help enhance the generalizability of the findings and provide a more comprehensive understanding of the factors influencing postpartum self-esteem, body appreciation, and breastfeeding outcomes. Additionally, future research should explore the long-term effects of breastfeeding support and self-esteem on mothers’ psychological well-being beyond the early postpartum period. It is also important to consider the cultural context of Cyprus in interpreting the findings. Cyprus has unique family structures and societal expectations around motherhood that may influence women’s experiences with breastfeeding and body image. Our study is the first in Cyprus to examine these particular relationships, and it highlights the need for culturally tailored interventions. By considering local cultural norms and expectations, healthcare professionals can better support mothers in managing both the challenges of breastfeeding and postpartum body image issues.

## 5. Conclusions

The findings from this study emphasize the need for further research on the factors that are related to breastfeeding as well as mothers’ body image and self-esteem. Longitudinal studies are particularly necessary to better understand the pathways that influence these relationships. In conclusion, the results underscore the critical role of breastfeeding support—both from healthcare professionals and partners—in improving mothers’ self-esteem and body appreciation. Addressing body image concerns during the postpartum period and offering comprehensive breastfeeding support are essential for enhancing mothers’ psychological well-being. Healthcare professionals should integrate these factors into their care practices, and (antenatal and postpartum) educational programs should focus on setting realistic expectations and highlighting the functional benefits of breastfeeding.

## Figures and Tables

**Table 1:** Bivariate Pearson’s r correlations among self-esteem and body appreciation at two days postpartum and pregnancy weight gain.

	1	2	3
1. Pregnancy Weight Gain	-		
2. Self-Esteem—at Two Days Postpartum	−0.293 **	-	
3. Body Appreciation—at Two Days Postpartum	−0.292 **	0.552 **	-

Note 1: ** Correlation is significant at the 0.01 level (2-tailed). Note 2: 1—Pregnancy Weight Gain, 2—Self-Esteem—at Two Days Postpartum, 3—Body Appreciation—at Two Days Postpartum.

**Table 2:** Multivariate analyses of variance of self-esteem and body appreciation at two days postpartum in relation to psychological influence of pregnancy weight gain.

	**Psychological Influence of Pregnancy Weight Gain**						
	Absent	Low	Moderate/High	***F***	***df***	***p***	***η* ^2^**
Self-Esteem—at Two Days Postpartum	2.35 ^a^	2.20 ^ab^	2.07 ^b^	4.390	2	0.015	0.083
Body Appreciation—at Two Days Postpartum	4.11 ^a^	4.04 ^ab^	3.54 ^b^	8.596	2	<0.001	0.151

Note: Means that do not share a common index (a, b) not differ significantly from each other according to the *Scheffé* post hoc criterion.

**Table 3:** Multivariate analyses of variance of level of acceptance of body changes in relation to self-esteem and body appreciation at two days postpartum.

	**Level of Acceptance of Body Changes**						
	Absent/ Low/Moderate	High	Very High	***F***	***df***	***p***	***η* ^2^**
Self-Esteem—at Two Days Postpartum	2.08 ^a^	2.22 ^ab^	2.38 ^b^	3.573	2	0.027	0.072
Body Appreciation—at Two Days Postpartum	3.51 ^a^	3.97 ^b^	4.27 ^b^	11.364	2	<0.001	0.190

Note: Means that do not share a common index (a, b) not differ significantly from each other according to the *Scheffé* post hoc criterion.

**Table 4:** Multivariate analyses of variance of self-esteem and body appreciation at two days postpartum in relation to breastfeeding difficulties.

	Breastfeeding Difficulties					
	No	Yes	***F***	***df***	***p***	***η* ^2^**
Self-Esteem—at Two Days Postpartum	2.32	2.12	4.755	1	0.032	0.055
Body Appreciation—at Two Days Postpartum	3.98	3.85	0.782	1	ns	0.009

Note: ns = nonsignificant.

**Table 5:** Bivariate Pearson’s *r* correlations among self-esteem and body appreciation at two days postpartum and breastfeeding support.

	1	2	3	4
1. Breastfeeding Support in Hospital	-			
2. Breastfeeding Support from Husband	0.506 **	-		
3. Self-Esteem—at Two Days Postpartum	ns	0.235 **	-	
4. Body Appreciation—at Two Days Postpartum	0.314 **	0.290 **	0.552 **	-

Note 1: ns = nonsignificant. Note 2: ** Correlation is significant at the 0.01 level (2-tailed).

**Table 6:** Linear regression of self-esteem and body appreciation at two days postpartum in relation to possible predictors.

	**Self-Esteem at Two Days Postpartum**				
	*b*	*S.E.*	*β*	*t*	*p*
(*Constant*)	2.469	0.231		10.705	<0.001
Pregnancy Weight Gain	−0.016	0.009	−0.205	−1.789	ns
Psychological Influence of Pregnancy Weight Gain	−0.093	0.045	−0.236	−2.088	0.040
Level of Acceptance of Body Changes	0.014	0.032	0.056	0.444	ns
Breastfeeding Difficulties	−0.238	0.088	−0.284	−2.711	0.008
Breastfeeding Support in Hospital	0.011	0.059	0.025	0.178	ns
Breastfeeding Support from Husband	0.031	0.049	0.083	0.630	ns
	*R* = 0.475, *R*^2^ = 0.226, *F* = 3.688, *df* = 6, *p* = 0.003				
	**Body Apritiation at Two Days Postpartum**				
	*b*	*S.E.*	*β*	*t*	*p*
(*Constant*)	3.753	0.338		11.111	<0.001
Pregnancy Weight Gain	−0.022	0.013	−0.172	−1.648	ns
Psychological Influence of Pregnancy Weight Gain	−0.166	0.065	−0.262	−2.550	0.013
Level of Acceptance of Body Changes	0.095	0.047	0.232	2.036	0.045
Breastfeeding Difficulties	−0.231	0.129	−0.171	−1.793	ns
Breastfeeding Support in Hospital	0.182	0.091	0.263	2.010	0.048
Breastfeeding Support from Husband	0.046	0.071	0.078	0.650	ns
	*R* = 0.599, *R*^2^ = 0.359, *F* = 7.100, *df* = 6, *p* < 0.001				

Note: ns = nonsignificant.

**Table 7:** Linear regression of degree of psychological influence of pregnancy weight gain in relation to influence of information on initiation and continuation of breastfeeding.

	*b*	*S.E.*	*β*	*t*	*p*
(Constant)	1.801	0.231		7.783	<0.001
Influence of Information on Initiation and Continuation of Breastfeeding	−0.278	0.087	−0.345	−3.207	0.002

Note: *R* = 0.354, *R*^2^ = 0.108, *F* = 10.283, *df* = 1, *p* = 0.002.

## Data Availability

The original contributions presented in this study are included in the article. Further inquiries can be directed to the corresponding author(s).

## Public Involvement Statement

No public involvement in any aspect of this research.

## Guidelines and Standards Statement

This manuscript was drafted for observational research.
